# The Impact of Alcohol Use Disorder on Tuberculosis: A Review of the Epidemiology and Potential Immunologic Mechanisms

**DOI:** 10.3389/fimmu.2022.864817

**Published:** 2022-03-31

**Authors:** Gregory W. Wigger, Tara C. Bouton, Karen R. Jacobson, Sara C. Auld, Samantha M. Yeligar, Bashar S. Staitieh

**Affiliations:** ^1^ Division of Pulmonary, Allergy, Critical Care and Sleep Medicine, Department of Medicine, Emory University School of Medicine, Atlanta, GA, United States; ^2^ Section of Infectious Diseases, Department of Medicine, Boston University School of Medicine, Boston, MA, United States; ^3^ Rollins School of Public Health, Emory University, Atlanta, GA, United States; ^4^ Atlanta VA Medical Center, Atlanta, GA, United States

**Keywords:** alcohol, alcohol use disorder (AUD), tuberculosis, alveolar macrophage (AM), innate immunity, oxidative stress

## Abstract

Globally, an estimated 107 million people have an alcohol use disorder (AUD) leading to 2.8 million premature deaths each year. Tuberculosis (TB) is one of the leading causes of death globally and over 8% of global TB cases are estimated to be attributable to AUD. Social determinants of health such as poverty and undernutrition are often shared among those with AUD and TB and could explain the epidemiologic association between them. However, recent studies suggest that these shared risk factors do not fully account for the increased risk of TB in people with AUD. In fact, AUD has been shown to be an independent risk factor for TB, with a linear increase in the risk for TB with increasing alcohol consumption. While few studies have focused on potential biological mechanisms underlying the link between AUD and TB, substantial overlap exists between the effects of alcohol on lung immunity and the mechanisms exploited by *Mycobacterium tuberculosis* (*Mtb*) to establish infection. Alcohol misuse impairs the immune functions of the alveolar macrophage, the resident innate immune effector in the lung and the first line of defense against *Mtb* in the lower respiratory tract. Chronic alcohol ingestion also increases oxidative stress in the alveolar space, which could in turn facilitate *Mtb* growth. In this manuscript, we review the epidemiologic data that links AUD to TB. We discuss the existing literature on the potential mechanisms by which alcohol increases the risk of TB and review the known effects of alcohol ingestion on lung immunity to elucidate other mechanisms that *Mtb* may exploit. A more in-depth understanding of the link between AUD and TB will facilitate the development of dual-disease interventions and host-directed therapies to improve lung health and long-term outcomes of TB.

## 1 Introduction

Alcohol misuse is a significant global health issue with wide-ranging and pervasive consequences. In 2016, 5.3% of all global deaths were attributable to alcohol consumption, and alcohol misuse was the 7^th^ leading risk factor for premature death and disability (1, 2). Alcohol use disorder (AUD) has been linked to an increased susceptibility to pulmonary infections and their associated complications for over 200 years (3). More recently, AUD has been found to be an independent risk factor for acute respiratory distress syndrome (ARDS) with a two to four-fold increased risk compared to individuals without AUD (4, 5). Similarly, persons with AUD have an increased risk for bacterial pneumonia and its associated morbidity and mortality (6–11). They also suffer from a higher incidence of serious complications from pneumonia, including bacteremia, parapneumonic effusion, and empyema (9, 12–14). AUD causes a variety of detrimental effects on the lungs including increased alveolar oxidative stress, immune impairments, and alterations in the metabolism of pulmonary cells.

Despite advances in diagnosis and treatment, tuberculosis (TB) is the second leading infectious killer worldwide, only recently surpassed by COVID-19 (15). While the global TB incidence rate has been decreasing annually since 2000, the most recent World Health Organization (WHO) global TB data reported an increase in TB mortality for the first time in 20 years, driven in large part by disruption of TB control programs from the COVID-19 pandemic (15).

The recognition of the association between alcohol and TB occurred even before the causative agent of TB was known. As early as the 19^th^ century, physicians noted the increased incidence of infections, like TB and other causes of pneumonia, among patients that consumed alcohol (3, 16). The co-occurrence of excessive alcohol intake and TB has been continually noted since that time. AUD is one of the most common global risk factors for TB, second only to undernutrition and, notably, ahead of HIV and smoking (15). In this critical review, we will parse the complex relationship between alcohol and TB. We highlight recent epidemiologic work demonstrating a direct relationship between alcohol misuse and TB. We discuss mechanisms by which alcohol causes lung injury and suppresses lung immunity *via* increases in oxidative stress and impairments to the pulmonary innate immune system. For each of these biological pathways impacted by alcohol, we will highlight the potential mechanisms that might favor *Mtb* infection and dissemination.

## 2 Alcohol and TB Epidemiology

**Figure 1:**
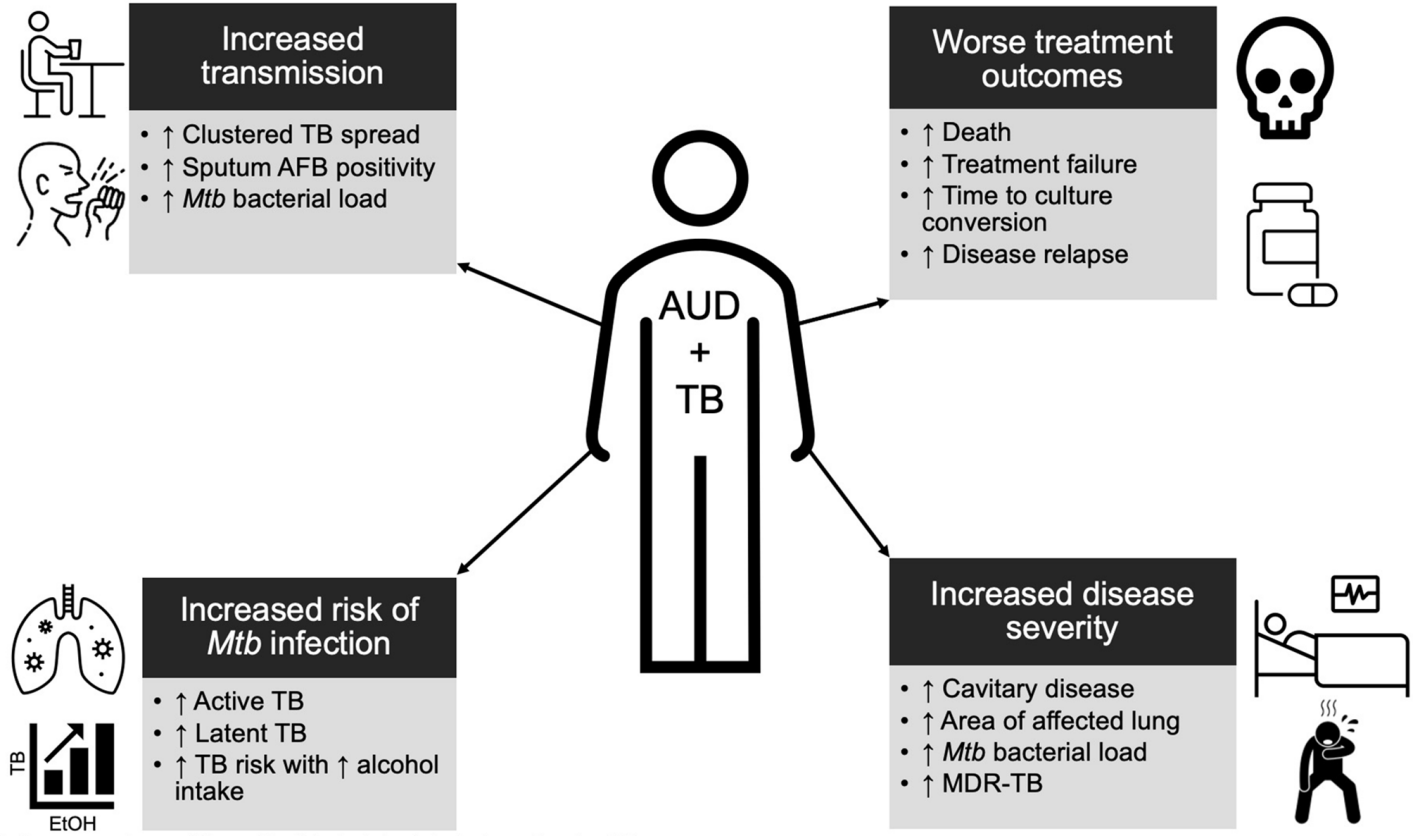
Summary schema of the epidemiologic data of alcohol use disorder (AUD) and tuberculosis (TB). Individuals with AUD are at a higher risk for TB, more infectious, have more severe disease, and are more likely to experience poor outcomes. See main text for further details.

Epidemiologic data support a clear relationship between alcohol and TB and reveal the various biologic, immunologic, and clinical impacts that alcohol may have on patients with TB (**Figure 1**).

### 2.1 Risk of Infection

The risk of both latent TB infection (LTBI) and active TB disease is higher among persons with alcohol use disorders (AUD) than those without AUD (17). For example, one study in New York City found a 28-fold higher rate of active TB disease among those with AUD as compared to age-matched individuals without AUD (18). Other studies have documented a dose-response relationship between active TB and alcohol consumption, with the risk of TB rising as a person’s daily alcohol consumption increases (19, 20).

While improvements in diagnostics and broader access to treatment have led to global declines in TB incidence since 2000 (21), cases of TB associated with AUD are on the rise, particularly among men (22, 23). It is estimated that 8-15% of global TB deaths are attributable to alcohol misuse and AUD (15, 20, 24). In high-income countries where non-communicable diseases, including diabetes and AUD, have a greater prevalence, over 35% of TB deaths among those under the age of 65 are linked to alcohol misuse (25).

Although the interactions between poverty, social marginalization, alcohol misuse, and TB remain complicated and difficult to disentangle, their overlap does not fully explain the increased risk of TB with AUD. Studies examining the relationship between AUD and TB have shown that AUD remains a significant independent risk factor for TB, with a relative risk of 2.9, even after controlling for confounders such as comorbidities, lifestyle, or social determinants of health (20, 24, 26–28).

### 2.2 Risk of Transmission and Severity

Individuals with TB and AUD are more infectious and have more clinically severe TB. Analyses of TB outbreaks have shown clustered TB spread and transmission among persons with AUD, and drinking venues and bars have been identified as sites of TB transmission (29–31). Further, molecular epidemiology studies have shown individuals with AUD are more likely to reflect recent TB transmission and to be part of a transmission cluster (32, 33). While this increased transmission may be due to the social marginalization often seen with AUD, it could also be due to an increased mycobacterial burden in persons with AUD. Cases of TB associated with AUD have higher rates of acid-fast bacilli (AFB) detected in sputum samples and increased *Mtb* bacterial load compared to TB without an AUD association (25, 26). Both of these characteristics have previously been associated with more severe TB features, including cavitation, as well as increased *Mtb* transmission and thus may explain some of the increased transmissibility of TB in AUD (25, 26, 34–36). Those with AUD and TB are more likely to present with pulmonary rather than extrapulmonary TB (25, 26). More of their lungs are affected by TB and they are predisposed to advanced, cavitary disease at the time of presentation (25, 26, 35, 37).

### 2.3 Treatment Outcomes

For those who initiate treatment, patients with TB and AUD have worse clinical outcomes, including increased time to culture conversion, incidence of TB treatment failure, rate of disease relapse, and risk of death (25, 28, 38–43). Again, the intersection of alcohol and certain social determinants complicates the interpretation of these data. For example, it is known that AUD has been associated with delays in accessing TB care and poor adherence to TB treatment, both of which may contribute to the more advanced, severe disease at the time of diagnosis and inadequate therapy (44, 45). More recent data indicate an increased incidence of multi-drug resistant TB in people with AUD, possibly due to poor treatment adherence as well as comorbidities associated with AUD that impact immune function and metabolism (43, 46, 47). However, as described above, poor TB outcomes persist among individuals with AUD even when controlling for behaviors that impact access to and retention in treatment (e.g., individuals with AUD lost to treatment follow-up) (28). A two- to four-fold increased risk of death remains for those with TB associated with AUD compared to those without AUD even when only considering patients being actively treated for TB (25, 48, 49).

### 2.4 Efficacy of Treatment

Alcohol-associated metabolic dysfunction and adverse drug effects have been a concern regarding individuals with AUD and TB. Studies have shown alcohol’s potential impact on the metabolism, absorption, and resultant concentrations of several TB drugs including isoniazid, rifampicin, and fluoroquinolones (50–58). Proper drug concentrations are essential for successful TB treatment. Sub-therapeutic concentrations are predictive of poor outcomes in TB, including death or disease relapse, while supra-therapeutic concentrations can lead to adverse events and treatment interruptions (59). Historically, such concerns have led to the exclusion of individuals with AUD from studies of TB preventative therapy. However, clinical trials are underway investigating the true benefit vs. harm of TB preventative therapy in persons with AUD (60). Alcohol intervention programs may also play an important role in the future of treatment for TB associated with AUD. Previous studies have shown a desire for such programs among TB patients, and the initiation of intervention programs led to favorable outcomes with improved treatment adherence in individuals with AUD and TB (61, 62).

## 3 Mechanisms of TB in AUD

### 3.1 Oxidative Stress

The lung’s constant exposure to the external environment and resultant processing of inhaled smoke, dust particles, microbes, toxins, etc. generates free radicals, including reactive oxygen species (ROS) and reactive nitrogen species (RNS), that are released into the alveolar environment. In the lungs, there are efficient antioxidant defense systems, including antioxidant enzymes and antioxidant stores, that defend against oxidants and other reactive species (63, 64). Maintaining a balanced oxidation-reduction (redox) state is essential for key cellular functions, such as proliferation, differentiation, and apoptosis (63). Redox balance can alter protein structure and reactivity as well as cell signaling pathways (65). These alterations can result in a release of inflammatory mediators and cytokines with subsequent macrophage activation, polymorphonuclear (PMN) cell recruitment, inflammation, and tissue damage (64). The redox state can be assessed by measuring thiol/disulfide couples including glutathione (GSH) and glutathione disulfide (GSSG), the primary thiol redox system within the alveoli (63). Epithelial lining fluid of a healthy lung has abundant extracellular GSH in order to detoxify oxidants and free radicals (63–65). Oxidative stress in the lung results when its antioxidant capacity is depleted. Both chronic alcohol ingestion as well as *Mtb* infection increase oxidative stress.

#### 3.1.1 Alcohol Increases Pulmonary Oxidative Stress

AUD increases pulmonary oxidative stress and induces an oxidized microenvironment within the lung through a variety of mechanisms. AUD depletes antioxidant stores, including GSH, within the pulmonary environment (66). GSH serves as the primary reducing agent in the alveolar space, acting as a substrate in a reaction with glutathione peroxidase that detoxifies peroxides in the lung including hydrogen peroxide and lipid peroxides (67). AUD alters pulmonary GSH homeostasis, resulting in an oxidation of GSH stores to form GSSG and oxidation of the GSH/GSSG redox potential (66, 68–70). Alcohol-induced depletion of GSH impairs an essential defense mechanism against oxidative stress in the lung.

There are a number of mechanisms through which alcohol depletes pulmonary GSH stores. First, alcohol induces mitochondrial dysfunction which decreases ATP generation and, in turn, may decrease GSH synthesis (68, 71). GSH can also be synthesized by reduction of GSSG, which utilizes NADPH as the electron donor. However, alcohol may reduce NADPH availability, resulting in decreased capacity for reduction and less GSH (71). Alcohol also increases ROS generation which then oxidizes GSH, further depleting GSH stores (71–73). A primary mechanism by which alcohol impairs GSH is its effects on the protein nuclear factor (erythroid-derived 2)-like 2 (Nrf2). Since Nrf2 is a transcription factor that activates hundreds of antioxidant genes and innate immune effectors (74), alcohol-mediated decreases in Nrf2 activation critically impairs the lung redox balance of those with AUD. Downstream effects from Nrf2 impairment include a diminished antioxidant response to oxidative stress and drained GSH stores (74).

AUD further increases alveolar oxidative stress by enhancing the expression and activity of NADPH oxidases (Nox). Nox proteins are membrane-associated enzymes that catalyze the reduction of molecular oxygen to superoxide and hydrogen peroxide, thus serving as major sources of ROS in the lungs. In alcohol-fed mice and rats, increased Nox expression increases alveolar oxidative stress (72, 73, 75). AUD depletes alveolar macrophage levels of peroxisome proliferator-activated receptor gamma (PPARγ) which, in turn, upregulates Nox proteins (76). Nox activity is likely further enhanced by the increase in TGFβ expression seen with AUD, which also upregulates particular Nox proteins (77, 78).

The increased oxidative stress within the alveolar microenvironment of individuals with AUD has important implications for pulmonary innate immunity. GSH deficiency increases alveolar epithelial intercellular permeability and diminishes surfactant synthesis (69, 79, 80). The oxidative stress associated with upregulated Nox protein expression impairs alveolar macrophage phagocytosis in alcohol-fed mice (72, 76). In environments of limited GSH, alveolar macrophage phagocytosis and microbe clearance are also compromised (68, 71). Supplementation of GSH has been demonstrated to restore alveolar macrophage function (71, 81, 82).

#### 3.1.2 *Mtb* Increases Pulmonary Oxidative Stress

Infection with *Mtb* has also been shown to increase oxidative stress and deplete antioxidant levels in the lungs. People with active TB disease had lower circulating levels of serum thiol and increased levels of its oxidized product serum disulfide (83). More broadly, mycobacterial infections, including *Mtb* and *Mycobacterium abscessus* (a rapid growing mycobacterium), have been associated with increased oxidative stress, as shown by decreases in total serum antioxidant capacity, total GSH, and increased lipid peroxidation (84–86). Treatment with antioxidants, including N-acetylcysteine (a precursor to GSH), have been shown to reduce oxidative stress as well as intracellular and pulmonary *Mtb* burden, while improving cell viability (84, 85).

Nrf2 is also an important aspect of the response to *Mtb*. Its expression is upregulated in *Mtb* infection due to the associated increase in ROS and oxidative stress (87, 88). Studies done *in vitro* with human macrophages have demonstrated benefit with pharmacologic Nrf2. Specifically, Nrf2 activation decreased oxidative injury, mitochondrial depolarization, and *Mtb*-induced ROS production in addition to inhibiting programmed necrosis of the macrophage (89).

An increase in oxidative stress, in the context of *Mtb* infection, allows for enhanced mycobacterial growth and survival. *Mtb* grows *in vitro* at a faster rate in more oxidized environments, as in the lung apices where clinical TB disease is most common (90). These observations are supported by a series of experiments with *M. abscessus* where increased intracellular growth was seen in more oxidized environments (86).

#### 3.1.3 Pulmonary Oxidative Stress: AUD and *Mtb* Overlap

Taken together, the co-occurrence of AUD and *Mtb* infection likely results in a significant increase in pulmonary oxidative stress state which benefits *Mtb*. First, the combination of oxidative stress and impairments in antioxidant defenses, particularly the depletion of GSH stores and Nrf2 inhibition by alcohol, may sufficiently derange immune function and facilitate *Mtb* infection and growth. Second, *Mtb*’s growth and survival improves in oxidative environments, making the oxidized alveoli in those with AUD a more ideal environment for *Mtb*. Collectively, alcohol-induced oxidative stress may generate the ideal combination for *Mtb* infection: an oxidized alveolus and an impaired host response.

### 3.2 Pulmonary Innate Immunity

The pulmonary innate immune system is a multilayered system for defense against pathogens, detection of tissue damage, and maintaining pulmonary tissue integrity and homeostasis (91). Chronic alcohol misuse exerts a wide range of effects on this system resulting in impairments of several vital functions of innate immunity (92, 93). Its adverse effects impact the basic defense and barrier functions of the cilia and alveolar epithelium as well as the functions of specialized cells including alveolar macrophages and PMNs. For example, AUD inhibits neutrophil margination, influx from the peripheral circulation into the alveolar space, and subsequent pathogen clearance and killing during infection (92, 94–96). Antigen-presenting cells (APCs) of the innate immune system, necessary for adaptive immune activation, have a decreased peripheral presence and impaired activity due to alcohol (97, 98).

Alongside the growing understanding of the negative impacts of alcohol, the understanding of *Mtb* infection and host-pathogen interactions is also evolving. With the increased occurrence and severity of TB with AUD, it is likely that *Mtb* exploits alcohol-mediated impairments in pulmonary innate immunity to establish infection and disseminate. Previous research has elucidated alcohol’s deleterious effects on pulmonary mucociliary function, alveolar epithelium, and the alveolar macrophage. Given this breadth of information, in addition to the alveolar macrophage being first line of defense in the alveolar space and the dominant cell type that *Mtb* infects, we will spend the remaining portion of this section reviewing the specific effects of chronic alcohol misuse on the pulmonary innate immunity and how these alcohol-mediated alterations may intersect with *Mtb* pathogenicity.

#### 3.2.1 Pulmonary Mucociliary Function

Ciliated airway cells are the first line of defense against inhaled pathogens and clear foreign particles from the lung. Chronic alcohol use repetitively exposes airways to ethanol eliminated from the bronchial circulation in exhaled breath. This repetitive injury impairs the integrity and function of airway cell cilia, ultimately leading to desensitization and resistance to motility, a phenomenon referred to as alcohol-induced ciliary dysfunction (99–101).

In the context of *Mtb*, this ciliary dysfunction facilitates transmission of airborne pathogens like *Mtb* into the lower airways, making it more likely that inhaled microbes will establish infection (102). Given that ciliary dysfunction has been associated with mycobacterial pulmonary infections, alcohol-induced ciliary dysfunction represents a likely mechanism by which AUD could predispose the host to *Mtb* infection (103).

#### 3.2.2 Alveolar Epithelium

Chronic alcohol use is also known to disrupt the pulmonary epithelial structure and function. AUD prevents the formation of a reliable, physical barrier of the alveolar epithelium by impairing tight junctions within its monolayer. Tight junctions are an important aspect of the epithelium as they closely associate cells and limit the passage of water, proteins, and other solutes across cell layers (104). Chronic alcohol ingestion alters the expression and interaction of essential components of the tight junctions, including claudin-1, claudin-5, claudin-7, occludin, and zonula occludens-1, resulting in a five-fold increase in pulmonary epithelial permeability (79, 105–107). People with AUD have increased alveolar-capillary permeability which predisposes them to the development of non-cardiogenic pulmonary edema compared to individuals without AUD (108, 109). Experiments in animal models support this mechanism of injury, with chronic alcohol consumption in rats increasing susceptibility to edematous lung injury (69). Alcohol also increases TGF-β1 expression while inhibiting granulocyte/macrophage colony-stimulating factor (GM-CSF) in the alveolar space, both of which have been implicated in disrupting and increasing the permeability of the alveolar epithelium (77, 110, 111). Lastly, AUD reduces the alveolar epithelial cell synthesis of surfactant, an important pattern recognition molecule that binds to various microbes and targets them for immune clearance (112, 113). Surfactant proteins have been shown to function as an opsonin that increases the *Mtb*-macrophage interaction and upregulates phagocytosis (114). Although the mechanism by which *Mtb* gains access to the lung interstitium from the alveolus is not fully understood, the putative mechanisms proposed in the literature include direct infection of alveolar epithelial cells and migration of *Mtb*-infected macrophages across the alveolar epithelium (115, 116). Both scenarios would be significantly more likely in the setting of alcohol-induced tight junction impairments and the more permeable alveolar epithelium of the alcohol-affected lung.

#### 3.2.3 Alveolar Macrophage

##### 3.2.3.1 Key Functions

The alveolar macrophage is essential for maintaining homeostasis of the lower airways through phagocytosis, removal of debris, and efferocytosis. It is also the first line of pulmonary immune defense in the alveolar space, responsible for recognizing, ingesting, and clearing pathogens, as well as release of cytokines and chemokines to recruit PMNs and monocytes to the site of invasion. Through a multitude of pathways and effects, AUD causes significant alveolar macrophage dysfunction, impairing phagocytosis, pathogen clearance, and cytokine release (71, 72, 76, 81, 82, 117–120).

AUD interferes with the maturation and terminal differentiation of the alveolar macrophage through inhibition of multiple signaling pathways. Alcohol interferes with GM-CSF signaling by downregulating GMCSF-Rβ expression on the alveolar macrophage surface resulting in impaired phagocytic function (117). Impairing GM-CSF signaling results in diminished expression of PU.1, a GM-CSF-dependent regulatory transcription factor required for normal alveolar macrophage cell development and differentiation as well as alveolar macrophage phagocytosis, pathogen killing, and cytokine production (118, 119, 121, 122). In addition to its previously mentioned role in oxidative defensive, Nrf2 additionally participates in alveolar macrophage maturation by increasing PU.1 expression by binding to its promoter region. Alcohol’s inhibition of Nrf2 has been shown to also be responsible for the decreased PU.1 expression of AUD (123).

*Mtb*’s interaction with the innate immune system is an ongoing area of interest and research. The alveolar macrophage is the primary cell that *Mtb* infects once it enters the lower respiratory tract (115, 116). Once phagocytosed by the alveolar macrophage, *Mtb* actively blocks phagosome maturation and fusion with the lysosome to ensure its survival and establish its intracellular niche (115, 124). In some cases, the alveolar macrophage is able to achieve successful intracellular killing of *Mtb*, likely through IFNγ and nitric oxide synthase signaling pathways (124). However, when *Mtb* evades killing, it replicates and eventually disrupts the phagosome membrane allowing *Mtb* into the alveolar macrophage cytosol for further replication. After infecting the alveolar macrophage, *Mtb* then gains access to the lung interstitium, where granuloma formation occurs (115). Subsequent host-pathogen interactions determine whether infection is cleared or if there is progression to latent TB infection or active TB disease, however a discussion of these later events is beyond the scope of this review (115).

GM-CSF is known to be an important for the innate immune response to mycobacterial infections, particularly with its role in restricting bacillary growth and promoting mycobacterial clearance (125–127). Patients with mycobacterial pulmonary infections have higher rates of GM-CSF signaling dysfunction compared to healthy controls (128). Further, treatment with recombinant GM-CSF (rGM-CSF) prior to mycobacterial infection enhanced intracellular killing and phagolysosomal fusion after *Mtb* infection as well as intracellular mycobacterial killing and superoxide anion release after *Mycobacterium avium complex* (MAC) infection (125, 129). Inhibiting GM-CSF by neutralizing GM-CSF antibodies prior to *Mtb* exposure dampens the proinflammatory cytokine release and neutrophil recruitment and increases *Mtb* burden in mouse macrophages (127). Thus, alcohol’s downregulation of GMCSF-Rβ on AMs and subsequent decreased GM-CSF expression likely contribute to an impaired response to *Mtb* infection.

Alcohol-induced impairments in innate immunity provide multiple avenues for facilitating *Mtb* infection. Data is limited on alcohol’s specific effects on the alveolar macrophage in the case of *Mtb* infection, however multiple studies have investigated other mycobacterial infections including MAC. Several studies done *in vitro* using human macrophages demonstrated that exposure to alcohol enhanced the intracellular growth of MAC and diminished the macrophage response to inflammatory cytokines (130, 131). Chronic exposure to alcohol also diminished macrophage production of bactericidal, innate immune effectors in response to MAC infection in a mouse model (132). Additional experiments showed increased dissemination, impaired pulmonary granuloma formation, and increased mycobacterial burden in alcohol-fed mice following mycobacterial infection (130, 133).

##### 3.2.3.2 Alveolar Macrophage Phenotype

Macrophages act as surveillance for the innate immune system, recognizing pathogens and tissue damage *via* pathogen-associated molecular patterns (PAMPs), damage-associated molecular patterns (DAMPs), and pattern recognition receptors. They have robust phagocytic and killing abilities and act as initiators of the inflammatory response. They function as antigen presenting cells that assist in the activation of the adaptive immune system. Further, macrophages participate in tissue repair, tissue remodeling, and maintain homeostasis (91). This plasticity in macrophage function occurs in response to surrounding physiologic state and cellular signaling and is referred to as macrophage phenotype (134).

Two paradigmatic states of the macrophage were initially described. The first being the “pro-inflammatory” or “classically activated” macrophage that responds to bacteria, viruses, lipopolysaccharide (LPS), and interferon gamma (IFNγ). It produces proinflammatory cytokines and chemokines like interleukin (IL)-12 and tumor necrosis factor alpha (TNFα), induces further inflammation and IFNγ release, and attracts neutrophils, natural killer (NK) cells, and lymphocytes to the site of infection (135). It relies heavily on glycolysis and fatty acid synthesis and a decrease in mitochondrial respiration (136). The second paradigmatic activation state is the “anti-inflammatory” or “alternatively activated” macrophage that is stimulated by IL-4 and participates in wound healing and tissue repair. It produces anti-inflammatory cytokines including IL-10 and IL-13 to reduce inflammation and promote tissue growth (135). Its metabolism is dependent on the tricarboxylic acid cycle (TCA) cycle and enhanced fatty acid oxidation (136). While macrophage differentiation was previously considered to be dichotomous and terminal, recent research suggests a more dynamic spectrum of activation and function. Depending on various extracellular signals, what were previously defined as all “anti-inflammatory” macrophages can exhibit dramatic differences in physiology, including overlap in function with some “pro-inflammatory” macrophages (137). Studies looking at gene expression of macrophages in pathologic conditions have demonstrated heterogenous activation and functionality as well as significant overlap in gene expression whether stimulated with LPS or IL-10 (138–140). Further data showed macrophage functional pattern changes with duration of stimuli and that they are capable of completely changing their phenotype based on the surrounding microenvironment (135, 141, 142). Overall, data collectively support the idea that macrophages are dynamic, plastic, and capable of displaying multiple, distinct, functional patterns.

Chronic alcohol exposure impacts alveolar macrophage functionality and makes phenotyping the alveolar macrophage in the context of alcohol misuse a complex issue (134). At baseline, alveolar macrophages isolated from animal models of chronic alcohol ingestion have increased IL-13 and TGF-β_1_ production, both associated with suppression of inflammation, as well as decreased phagocytic ability (77, 78, 143). These findings are likely due, at least in part, to alcohol-induced oxidative stress in the alveolar environment (as discussed previously). However, when exposed to alcohol and PAMPs, such as LPS, alveolar macrophages exhibit an exaggerated inflammatory response. In studies utilizing AMs from subjects with AUD, alveolar macrophages produce increased proinflammatory cytokines, including TNFα, IFNγ, IL-1β, and IL-6, in response to LPS stimulation compared to persons without AUD (144–146). Despite this elevation in inflammatory signals, multiple studies note a persistent decrease in alveolar macrophage phagocytosis when exposed to bacterial pathogens or PAMPs (72, 76, 117). This overexuberant response to pathogen stimulation has been postulated to be a contributing factor in the elevated risk for disproportionate inflammatory states, like ARDS, in people with AUD (147). While some of these phenotypic changes are related to the alcohol-induced oxidative stress in the lung, chronic alcohol exposure also alters alveolar macrophage metabolism (76, 82). AUD been shown to impair LPS-induced glycolytic response and induce mitochondrial derangements in the alveolar macrophage which can alter its cytokine response and contribute to phagocytosis impairments (148–150).

*Mtb* has also been noted to induce phenotypic changes in the alveolar macrophage after infection. In the early stages of infection, *Mtb* induces a robust production of inflammatory cytokines, increased phagocytosis, and upregulation of glycolysis from the alveolar macrophage (151–155). Glycolysis is important to the immune response because glycolytic inhibition results in an increased mycobacterial burden (156). Once intracellular, *Mtb* itself attempts to evade the macrophage’s killing processes by secreting virulence factors that inhibit the alveolar macrophage’s expression of the nuclear factor-κB (NF-κB) and IFNγ, ultimately promoting *Mtb*’s intracellular survival (151, 157). Following the initial response to *Mtb*, alveolar macrophages increase production of anti-inflammatory cytokines, including IL-10 and TGF-β, with decreased glycolysis and increased oxidative phosphorylation and free fatty acid metabolism (151, 158–162). Successful treatment of TB leads to resolution of these phenotypic changes to the macrophage (163).

Further work is clearly necessary to fully understand the relationship between AUD, *Mtb*, and alveolar macrophage function. The quiescent, baseline state of alveolar macrophages in subjects with AUD with increased expression of TGF-β_1_ may provide more favorable conditions for *Mtb* infection and facilitate intracellular proliferation. Further, glycolysis is integral to the macrophage’s response to *Mtb*: a decreased glycolytic reserve has been associated with *Mtb* infection and risk factors for TB (156, 164). Alcohol’s impairments of LPS-induced glycolysis may contribute to alveolar macrophage dysfunction and increase the risk of *Mtb* infection in those with AUD.

## 4 Conclusion and Future Directions

**Figure 2:**
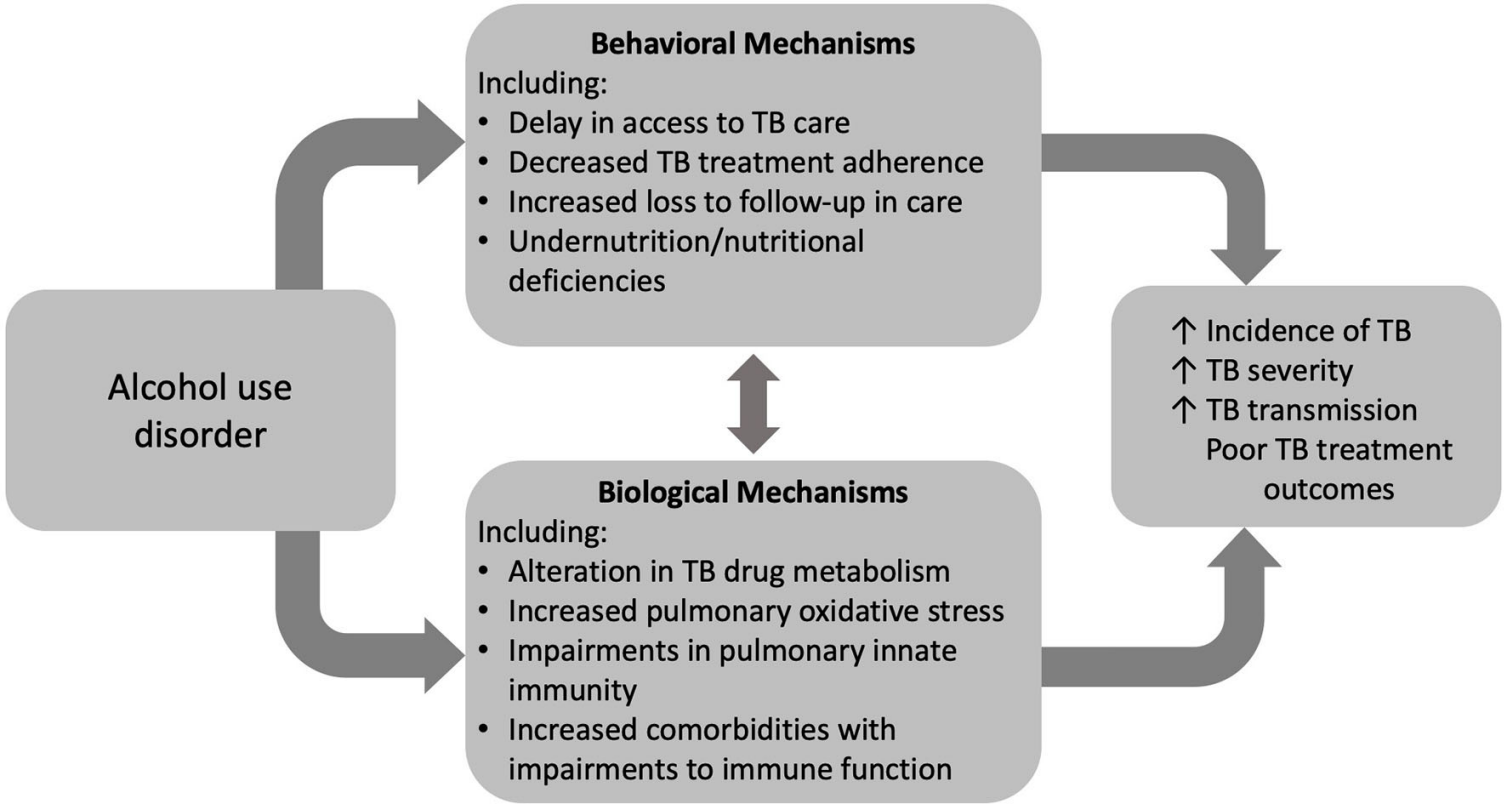
Alcohol use disorder (AUD) influences tuberculosis (TB) care and outcomes through both behavioral and biologic mechanisms.

**Figure 3:**
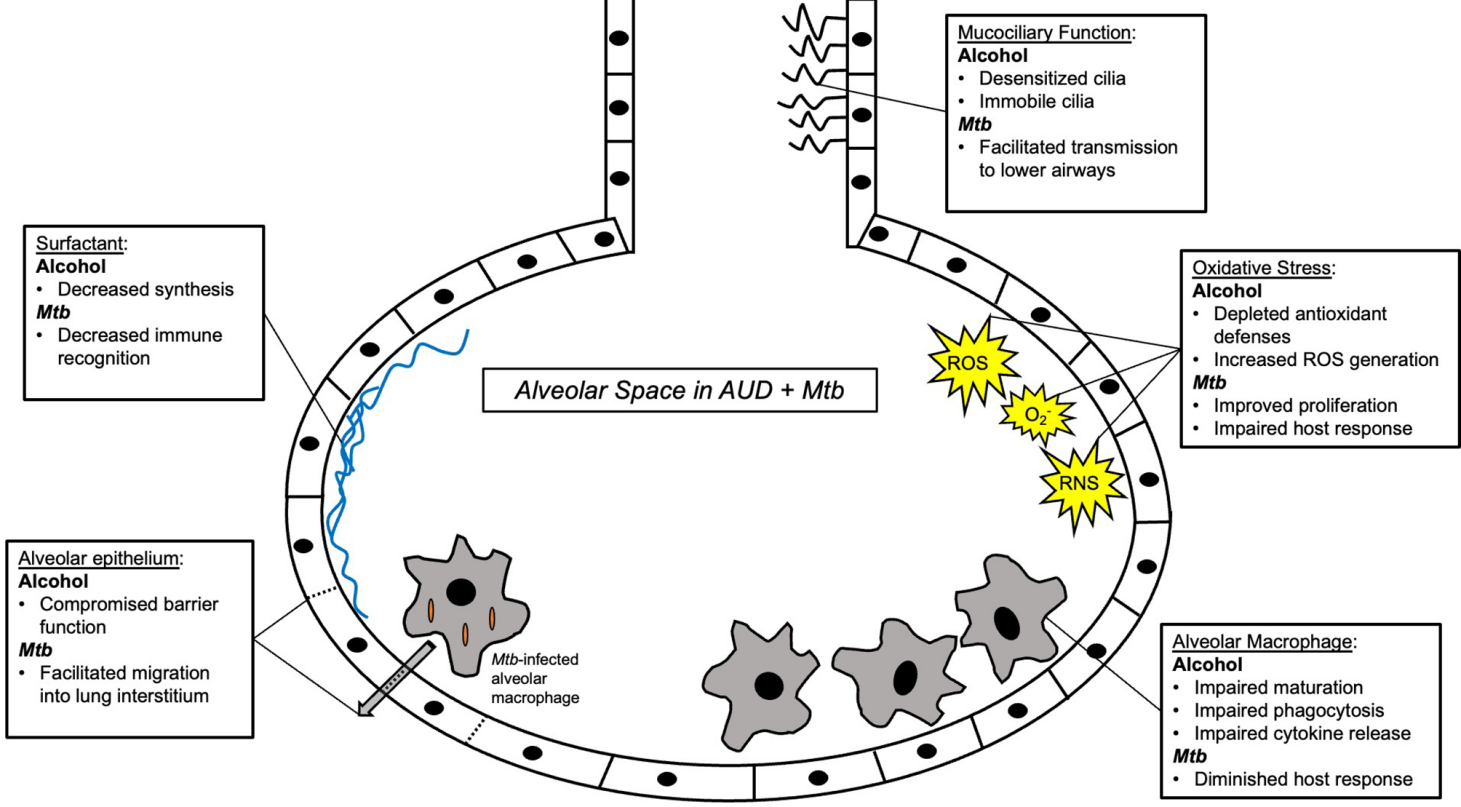
Summary schema of the alveolar space in alcohol use disorder (AUD) and Mycobacterium tuberculosis (Mtb) infection. Multiple components of innate immunity are affected by AUD, including macrophages, surfactant, the alveolar epithelium, and the alveolar oxidative state. See main text for further details.

Increasing rates of AUD pose a significant barrier to reaching the global goal of TB elimination. AUD is a risk factor for TB infection, severe disease, transmission, and associated death. Despite behavioral determinants of health linking AUD and TB disease, compelling evidence supports a biological impact of alcohol on TB risk and disease (**Figure 2**). The impact of alcohol on oxidative stress in the alveolar environment as well as impairments to the alveolar epithelium, alveolar macrophage, and remainder of the pulmonary innate immune system may facilitate *Mtb* infection and evasion of host defenses (**Figure 3**). To date, research directly investigating the mechanistic causes of TB in persons with AUD has been limited. Future investigations into the roles of innate immunity and oxidative stress specifically in alcohol and TB are needed. A better understanding of these causal pathways will lead to the development of host-directed therapies and better treatment outcomes in individuals with AUD and TB.

## Author Contributions

Design and conception - GW. Drafting and revising manuscript – GW, TB, KJ, SA, SY, and BS. All authors contributed to the article and approved the submitted version.

## Funding

This study was funded by the National Heart, Lung, and Blood Institute [T32 HL116271], the National Institute of Allergy and Infectious Diseases (NIAID) [K23 AI152930; R01 AI119037; K23 AI134182], and the National Institute on Alcohol Abuse and Alcoholism (NIAAA) [R01 AA026086; K08 AA024512].

## Conflict of Interest

The authors declare that the research was conducted in the absence of any commercial or financial relationships that could be construed as a potential conflict of interest.

## Publisher’s Note

All claims expressed in this article are solely those of the authors and do not necessarily represent those of their affiliated organizations, or those of the publisher, the editors and the reviewers. Any product that may be evaluated in this article, or claim that may be made by its manufacturer, is not guaranteed or endorsed by the publisher.
